# Relationship Between Navigation Success, Diagnostic Accuracy, and Ventilation Strategy: Retrospective Chart Review of 224 Consecutive Navigational Bronchoscopic Procedures Performed Under General Anesthesia †

**DOI:** 10.3390/jcm15041569

**Published:** 2026-02-16

**Authors:** Basavana Goudra, Prarthna Chandar, Divakara Gouda, Harrison Yang, Ganan Muhunthan, Suvan Sundaresh, Michael Green

**Affiliations:** 1 Department of Anesthesiology, Thomas Jefferson University, 111 S 11th Street, #7132, Philadelphia, PA 19107, USA; michael.green2@jefferson.edu; 2 Department of Pulmonary, Allergy and Critical Care, Sidney Kimmel Medical College, Philadelphia, PA 19107, USA; prarthna.chandar@jefferson.edu; 3 Department of Medicine, Inspira Health Network, 155 Bridgeton Pike Suite C, Mullica Hill, NJ 08062, USA; goudad1@ihn.org; 4 Sidney Kimmel Medical College, Department of Medicine, Thomas Jefferson University, 1100 Walnut St, Philadelphia, PA 19107, USA; harrison.yang@students.jefferson.edu (H.Y.); ganan.muhunthan@students.jefferson.edu (G.M.); suvan.sundaresh@students.jefferson.edu (S.S.)

**Keywords:** navigational bronchoscopy, anesthesia, ventilation, recruitment maneuver, atelectasis, pulmonary nodules

## Abstract

**Background**: Navigational bronchoscopy (NB) enables precise sampling of peripheral and central pulmonary nodules using shape-sensing or electromagnetic guidance. A major challenge is anesthesia-induced atelectasis, which alters lung anatomy, reduces registration accuracy, and is known to lower diagnostic accuracy. To counteract this, ventilatory protocols such as the Ventilatory Strategy to Prevent Atelectasis (VESPA) and the Lung Navigation Ventilation Protocol (LNVP) have been recommended. Their adoption and clinical impact, however, remain uncertain. **Methods**: We conducted a retrospective review of 224 consecutive NB procedures performed under general anesthesia at a single academic medical center (January 2020–August 2024). Demographic, anesthetic, and ventilatory data were extracted from electronic records. Outcomes included navigational success (ability to reach the lesion) and diagnostic accuracy (concordance between bronchoscopic diagnosis and final clinical diagnosis after follow-up). Ventilatory practices were compared with published VESPA and LNVP recommendations. **Results**: Navigational success, defined as successful advancement of the bronchoscope to the target lesion with tissue acquisition, was achieved in 89.2% of cases. Overall diagnostic accuracy, defined as concordance between bronchoscopic diagnosis and final clinical diagnosis after follow-up, was 81.7%. Ventilatory management consistently diverged from recommended protocols. Most patients were ventilated with FiO_2_ > 0.6, PEEP in the range of 7–10 cm H_2_O, and tidal volumes of 300–500 mL. The only recommended maneuver systematically applied was recruitment immediately after intubation. Despite widespread deviation from both VESPA and LNVP, diagnostic performance remained favorable relative to published benchmarks. No major anesthesia-related complications occurred. **Conclusions**: In this retrospective series, navigational success comparable to published studies that adapted strict ventilation protocols was achieved with also comparable diagnostic accuracy without strict adherence to predefined ventilatory strategies. Recruitment maneuvers may represent the most influential component of current protocols, but institutional factors such as procedural expertise and case volume likely contributed to outcomes. Prospective studies are warranted to determine whether standardized ventilatory protocols are necessary for optimizing NB performance.

## 1. Introduction

Navigational bronchoscopy (NB), with or without robotic assistance, is a novel technique uniquely suited for sampling both peripheral and central pulmonary nodules. Depending on the platform, NB may use either electromagnetic field guidance or shape-sensing technology. This approach is associated with a high diagnostic accuracy and low complication rates [1]. The procedure typically begins with a pre-procedural chest CT, from which a three-dimensional virtual airway map is constructed. Using this map, the bronchoscopist navigates to the target lesion, after which biopsy and/or interventions such as fiducial/dye marking may be performed prior to surgery.

In addition to lesion-specific factors—such as size, location (upper vs. lower lobes), presence of a bronchus sign, and registration accuracy—anesthesia management plays an important role in procedural success. The registration accuracy refers to how precisely the patient’s actual anatomy (as seen by the bronchoscope or tracked by sensors) aligns with the pre-procedural imaging dataset (usually a CT scan) used for navigation. If the virtual CT map and the actual bronchoscope location match very closely, it is easier to reach peripheral nodules or small targets reliably. Anesthesiologists must provide optimal conditions to facilitate accurate navigation and successful tissue sampling. A key challenge is that pre-procedural CT scans, on which NB relies, are acquired in awake, spontaneously breathing patients. Under general anesthesia, pulmonary atelectasis can occur, especially in the lower lobes, resulting in CT-to-body divergence (spatial mismatch between the CT model and the true airway anatomy), thereby obscuring the target lesion on intraprocedural cone-beam CT images. This phenomenon can compromise both targeting precision and diagnostic accuracy [2].

To mitigate these effects, anesthesia providers are advised to follow specific ventilatory strategies. Two commonly cited protocols are the Ventilatory Strategy to Prevent Atelectasis (VESPA) and the Lung Navigation Ventilation Protocol (LNVP) [3]. VESPA recommendations include: volume-controlled ventilation, tidal volumes of 6–8 mL/kg ideal body weight (IBW), the lowest FiO_2_ required to maintain SpO_2_ ~94%, PEEP of 8–10 cm H_2_O, and a recruitment maneuver immediately after intubation (10 consecutive breaths at a plateau pressure of 40 cm H_2_O with PEEP of 20 cm H_2_O in pressure control mode) [2,4]. LNVP, by contrast, recommends larger tidal volumes (10–12 mL/kg IBW), the lowest tolerable FiO_2_, and higher PEEP settings—10–15 cm H_2_O for upper/middle lobe lesions and 15–20 cm H_2_O for lower lobe lesions. Recruitment maneuver with sustained inflation is employed at 30–40 cm H_2_O for 20–30 s

Despite increasing adoption of ventilatory protocols such as VESPA and LNVP, real-world adherence and their true clinical impact on navigational success and diagnostic accuracy remain uncertain. In many centers, anesthetic management is influenced by institutional workflow, operator experience, and competing physiological priorities rather than strict protocolized ventilation strategies. We therefore undertook this study to evaluate real-world ventilatory practices during navigational bronchoscopy at our institution and examine whether deviation from recommended protocols influenced navigational success or diagnostic accuracy.

## 2. Materials and Methods

### 2.1. Study Design and Setting

This was a single-center, retrospective cohort study conducted at Thomas Jefferson University Hospital (Philadelphia, PA, USA). The study was approved by the Institutional Review Board (IRB) of Thomas Jefferson University (protocol number iRISID-2024-0996). The requirement for informed consent was waived due to the retrospective nature of the investigation.

### 2.2. Patient Selection

We identified all consecutive adult patients (≥18 years) who underwent navigational bronchoscopy (NB) between January 2020 and August 2024.

Inclusion criteria: Patients undergoing NB for evaluation of pulmonary nodules or masses, performed under general anesthesia with an endotracheal tube.

Exclusion criteria: Patients with incomplete anesthesia records (missing ventilatory parameters), procedures performed without navigation assistance, or cases with aborted procedures due to non-pulmonary reasons (e.g., hemodynamic instability, technical failure).

### 2.3. Data Sources and Abstraction

Data were abstracted from the electronic medical record (Epic Systems Corporation, Verona, Wisconsin, USA). The data were collected and entered by the medical students under the supervision of the principal investigator in a standardized data collection excel sheet that was used to capture variables, including: 

Demographics: Age, sex, height, weight, BMI, and ASA physical status.

Comorbidities: Hypertension, coronary artery disease, ischemic heart disease, pulmonary hypertension, OSA, and prior bronchoscopy.

Anesthetic management: Induction and maintenance agents, doses of propofol and fentanyl, use of neuromuscular blockade, airway device type and size, and duration of anesthesia and procedure.

Ventilatory parameters: Mode of ventilation (volume vs. pressure control), tidal volume (mL and mL/kg ideal body weight), PEEP (cm H_2_O), FiO_2_ (%), use of recruitment maneuvers, and end-tidal CO_2_ levels. Values were extracted at standardized time points (after induction/intubation, every 15 min and at completion).

Procedural details: Lesion location (right, left, bilateral) and use of adjunct imaging (cone-beam CT, fluoroscopy). Procedures were performed using an Ion Endoluminal System (Intuitive Surgical Inc., Sunnyvale, CA, USA) for robotic-assisted navigation. An Olympus T190 bronchoscope (Olympus Medical Systems Corp., Tokyo, Japan) was used for initial airway evaluation and post-procedure airway inspection.

Pathology results: Diagnostic category (malignant, benign, suspicious/atypical, nondiagnostic). 

### 2.4. Outcome Definitions

Navigational success: Defined as successful advancement of the bronchoscope and/or instruments to the target lesion as documented in the procedure note, confirmed by radial EBUS visualization and, when required, adjunct cone-beam CT imaging.

Diagnostic accuracy: Defined as the proportion of NB procedures resulting in a definitive pathological diagnosis (malignant or benign, including infectious or inflammatory etiologies). “Negative for malignancy” was considered diagnostic if an alternate accepted non-malignant diagnosis was established. Suspicious/atypical results were initially categorized as nondiagnostic; final classification after follow-up reclassified cases as true or false based on clinical outcome.

### 2.5. Statistical Analysis

Descriptive statistics were used to summarize patient demographics, anesthetic characteristics, ventilatory parameters, and procedural outcomes. Continuous variables are presented as medians with interquartile ranges (IQRs), while categorical variables are expressed as counts and percentages.

Ventilatory settings—including fraction of inspired oxygen (FiO_2_), positive end-expiratory pressure (PEEP), and tidal volume (mL and mL/kg ideal body weight)—were analyzed at standardized time intervals (immediately after induction, every 15 min, and at procedure completion). Data were visualized using two-dimensional histograms and three-dimensional scatter plots to illustrate the distribution of ventilation parameters and their relationship to diagnostic outcomes. Temporal trends of FiO_2_, PEEP, and tidal volume were evaluated to assess variability and compliance throughout the procedure.

Protocol adherence was assessed by comparing intraoperative ventilatory parameters against established criteria for the Ventilatory Strategy to Prevent Atelectasis (VESPA) and the Lung Navigation Ventilation Protocol (LNVP). A case was considered adherent if ≥90% of recorded values remained within the target range for a given protocol. Cases that did not meet these criteria were categorized as non-protocolized. Recruitment maneuvers were analyzed separately based on documentation in the anesthesia record.

Given the retrospective and descriptive nature of the study, no formal hypothesis testing or inferential statistical analyses were performed. The primary objective was to characterize patterns of anesthetic and ventilatory management, determine the degree of adherence to recommended protocols, and explore their association with navigation success and diagnostic accuracy through descriptive and graphical methods.

All data were organized and analyzed using spreadsheet-based tools, with graphical visualization performed using standard data visualization software.

### 2.6. Protocol Adherence Assessment

To evaluate compliance with proposed ventilatory protocols, we compared recorded intraoperative ventilatory settings against published recommendations for the Ventilatory Strategy to Prevent Atelectasis (VESPA) and the Lung Navigation Ventilation Protocol (LNVP). Components of both are described in the Section 1.

### 2.7. Operationalization

For each case, the recorded tidal volume, FiO_2_, and PEEP values were extracted from anesthesia records at standardized intervals (post-intubation, every 15 min, completion).

If ventilatory settings remained within the recommended range for a given protocol during most of the procedure (≥90% of recorded time points), the case was considered to have followed that protocol.

If settings did not consistently meet criteria for either protocol, the case was categorized as “non-protocolized.”

Recruitment maneuvers were documented separately, as these are often noted explicitly in anesthesia flowsheets or procedural records.

## 3. Results

### 3.1. Patient Characteristics and Anesthetic Management

A total of 224 navigational bronchoscopy procedures were analyzed, demonstrating high navigational success (89.2%) and diagnostic accuracy (81.7%) despite substantial variability and frequent deviation from recommended ventilatory protocols.

Table 1 summarizes the baseline demographic information and relevant clinical history. A total of 224 navigational bronchoscopy procedures were included in the study. The cohort consisted of 88 male and 136 female patients. The median height was 1.65 m (IQR: 0.15), median weight was 69.7 kg (IQR: 23.8), and median body mass index (BMI) was 25.1 (IQR: 6.8).

Most patients were classified as ASA physical status III (*n* = 168), followed by ASA II (*n* = 33) and ASA IV (*n* = 23). The most common comorbidity was hypertension (*n* = 114), followed by coronary artery disease (*n* = 30), obstructive sleep apnea (*n* = 30), ischemic heart disease (*n* = 11), and pulmonary hypertension (*n* = 9). A total of 32 patients had a prior history of bronchoscopy.

Table 2 provides the procedure and anesthesia information. Most bronchoscopies were performed in the right lung (40%) or bilaterally (37.3%), with fewer in the left lung (21.8%).

Propofol was the main anesthetic agent with a median induction dose of 150 mg and a total dose around 180 mg. Fentanyl was almost uniformly dosed at 100 µg. Procedures typically lasted about 82 min, while overall anesthesia time was longer (127 min). Most patients were intubated with an 8.5 mm endotracheal tube (90.1%).

### 3.2. Diagnostic Outcomes and Classification

Of 224 procedures, complete long-term diagnostic classification was available for 213 cases; these were included in diagnostic performance analysis. As demonstrated in Table 3, final diagnostic classification demonstrated 95 true positives, 79 true negatives (45 specific benign diagnoses and 34 nonspecific benign diagnoses), 4 false positives, 12 false negatives, and 23 nondiagnostic procedures. Among the characterized non-malignant lesions, the most common diagnoses included organizing pneumonia, granulomatous inflammation, and hamartomas, with additional microbiological findings consistent with infectious etiologies in select cases. Definitive etiologic diagnosis was not pursued for all benign lesions when not required for clinical management. Navigational success was achieved in 190 of 213 cases (89.2%). Overall diagnostic accuracy was 81.7%, calculated as (true positives + true negatives) divided by total evaluable cases (N = 213). Diagnostic accuracy of 81.7% compares favorably with pooled meta-analysis estimates (~70–75%). Given that navigational success remained high (89.2%) despite variability in ventilatory management, the impact of these ventilatory settings on procedural outcomes appears unlikely to be a dominant determinant of procedural outcomes in this cohort.

### 3.3. Lesion Characteristics

Lesion characteristics and distribution are summarized in Table 4. Target lesions were at least 4 mm in diameter, with many substantially larger and deemed clinically appropriate for biopsy. The majority of lesions were located in the upper lobes, with approximately one-third arising from lower lobes.

### 3.4. Ventilatory Management

**Figure 1:**
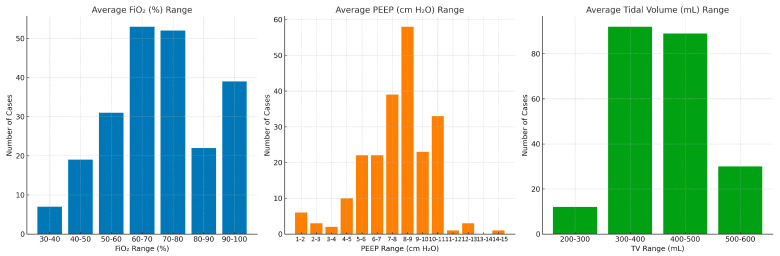
Ventilator Settings Bar Charts.

As illustrated in Figure 1, the ventilatory settings deviated considerably from the VESPA and LNVP recommendations.

The figure highlights the following observations:

- Left Panel (FiO_2_ % Range): Most cases were managed with FiO_2_ between 60-70% and 70–80% (over 50 cases each). A substantial number also required 90-100% FiO_2_ (~40 cases), while relatively few were in the lower ranges (30-40% and 40-50%), indicating a predominant reliance on higher FiO_2_ levels. This is quite contrary to the recommendations.
- Middle Panel (PEEP, cm H_2_O): The majority of patients received PEEP in the 8–9 cm H_2_O range (~58 cases). Additional clusters were noted in the 7–8 cm H_2_O and 10–11 cm H_2_O ranges. Only a few cases employed very low PEEP (1–4 cm H_2_O) or very high PEEP (>12 cm H_2_O), suggesting practice was centered on moderate PEEP settings (7–10 cm H_2_O).
- Right Panel (Tidal Volume, mL): Most patients were ventilated with tidal volumes in the 300–400 mL and 400–500 mL ranges (nearly 90 cases each). Smaller numbers were observed at the extremes (200–300 mL and 500–600 mL), reflecting a concentration of practice within the lung-protective range (300–500 mL).

**Figure 2:**
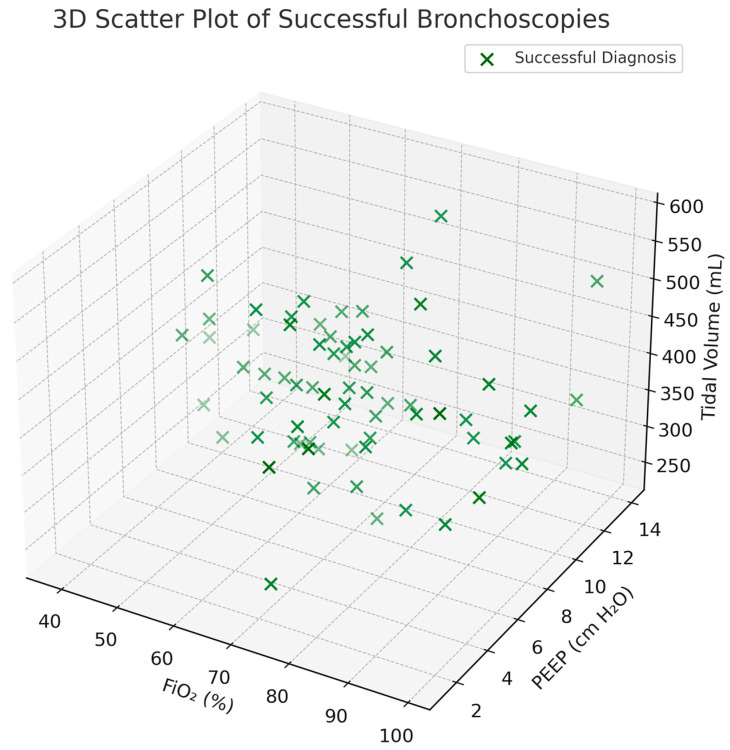
Successful Bronchoscopies Scatter Plot.

Figure 2 is a 3D scatter plot of successful bronchoscopies, illustrating the distribution of diagnoses in relation to three ventilatory parameters: FiO_2_ (%) on the *x*-axis, PEEP (cm H_2_O) on the *y*-axis, and tidal volume (mL) on the *z*-axis. Each green “×” represents a case with a successful bronchoscopy diagnosis. Most procedures clustered around FiO_2_ levels of 60–80%, PEEP values of 6–10 cm H_2_O, and tidal volumes between 300 and 450 mL. Very few patients received FiO_2_ >90% or extreme PEEP values (<4 or >12 cm H_2_O). Nevertheless, the use of excessively high FiO_2_ (>90%) or extreme PEEP values (<4 or >12 cm H_2_O) was uncommon and likely unnecessary.

**Figure 3:**
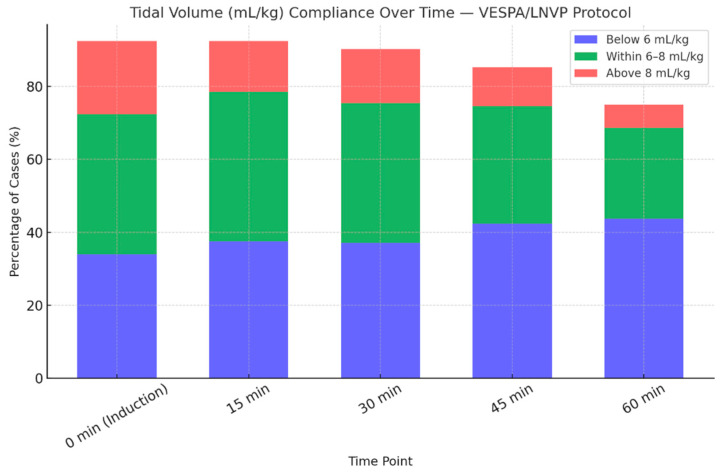
Tidal volume compliance over time. This figure illustrates the proportion of patients ventilated with tidal volumes outside the recommended range, assessed at 15 min intervals throughout the procedure.

**Figure 4:**
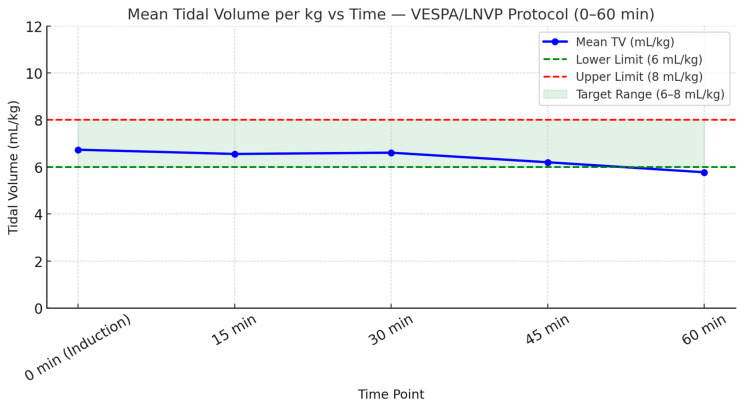
Mean tidal volumes over the first 60 min. This figure presents the mean tidal volumes recorded at 15 min intervals during the first hour. While the graph suggests that compliance was largely maintained, Figure 3 reveals that individual deviations from recommended tidal volumes were more frequent than the mean values indicate.

**Figure 5:**
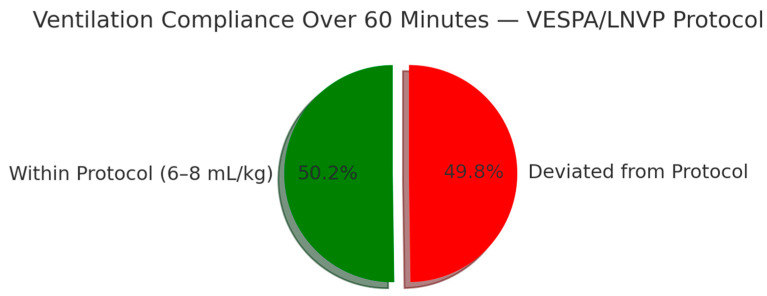
Overall compliance with ventilation protocols. This figure depicts the overall adherence to either of the recommended ventilation protocols throughout the study period.

Tidal volumes, measured at 15 min intervals from induction, were adjusted for body weight and assessed against VESPA/LNVP limits. As shown in Figure 3 and Figure 4, tidal volumes exhibited a time-dependent deviation from the recommended range. During the first 60 min of anesthesia, approximately half of all measurements were outside protocol limits, indicating variable compliance. Figure 5 further illustrates that tidal volumes remained beyond the target range for nearly half of the monitored period. The deviation of mean tidal volumes across all patients might appear acceptable; however, it is deceptive.

**Figure 6:**
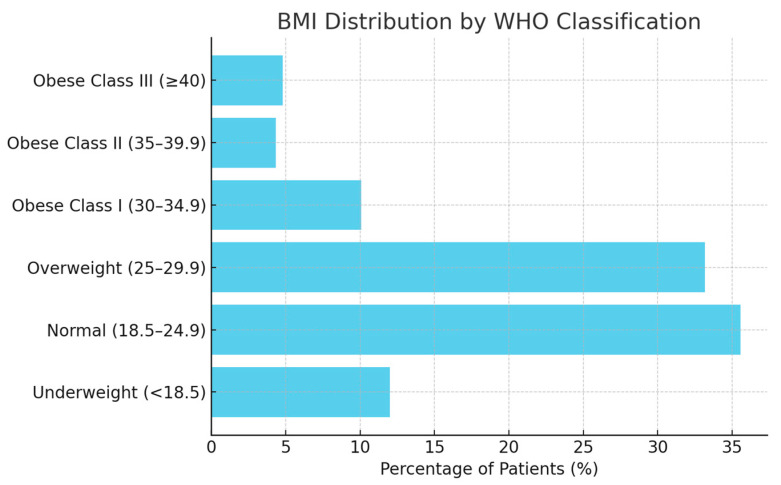
BMI distribution by WHO classification. This figure illustrates the distribution of patients across BMI categories as defined by the World Health Organization (WHO).

The average BMI was 26.1 kg/m^2^ (Overweight). A small percentage were underweight (Table 5). However, as can be seen in Figure 6, approximately 19.2% of patients in your dataset were classified as obese according to WHO criteria.

**Figure 7:**
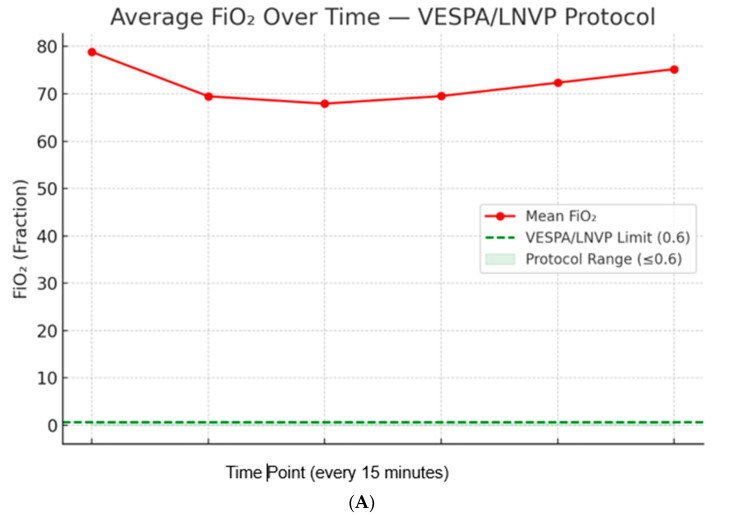

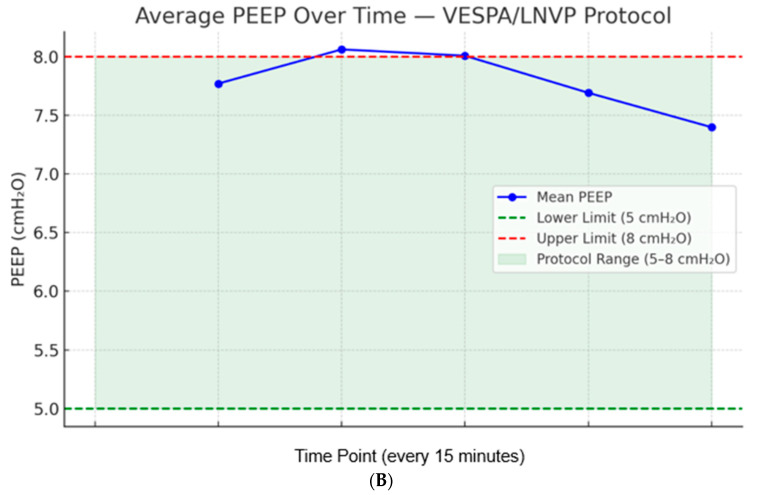
(**A**) Average FiO_2_ over time (15 min intervals). This figure shows the mean FiO_2_ values recorded every 15 min. When interpreted alongside Figure 9, it suggests that the elevated FiO_2_ levels were not intentionally used to maintain SpO_2_ around 94%. (**B**) Average PEEP over time (15 min intervals).

**Figure 8:**
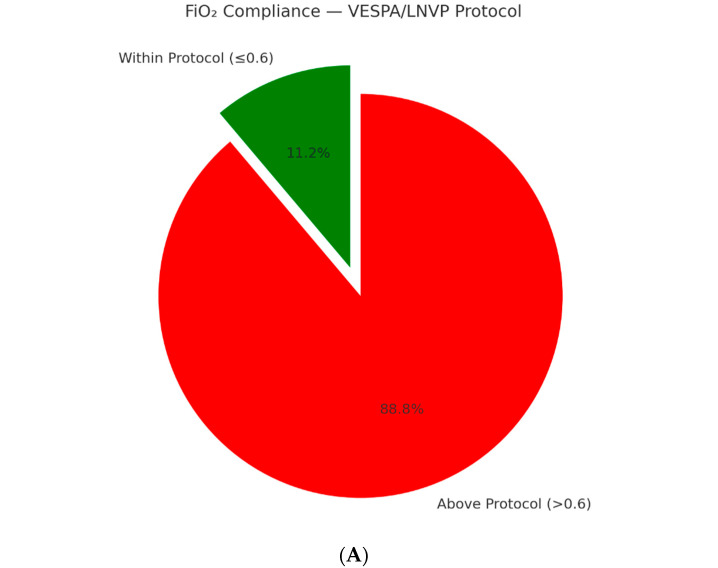

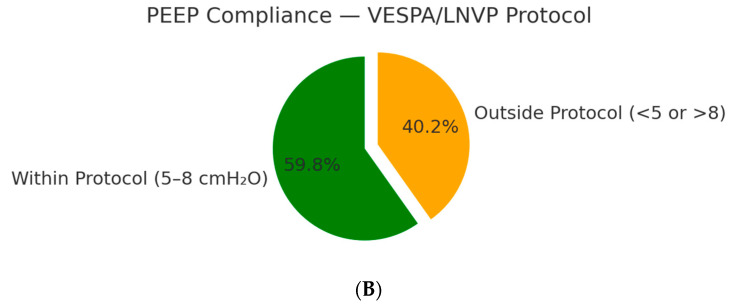
(**A**) This figure demonstrates poor compliance with the FiO_2_ requirements outlined in the VESPA/LNVP. (**B**) This figure demonstrates poor compliance with the PEEP requirements outlined in the VESPA/LNVP.

Figure 7A,B better explain the deviation of FiO_2_ from the protocol. Analysis of first 60 min of anesthesia shows that only 11.2% of cases were within the target (≤60%) and 88.8% were outside protocol, with most patients receiving much higher FiO_2_. As can be seen in Figure 8, PEEP compliance with LNVP/LNVP was 59.8%. Figure 7A,B provides average FiO_2_ over time and average PEEP over time.

**Figure 9:**
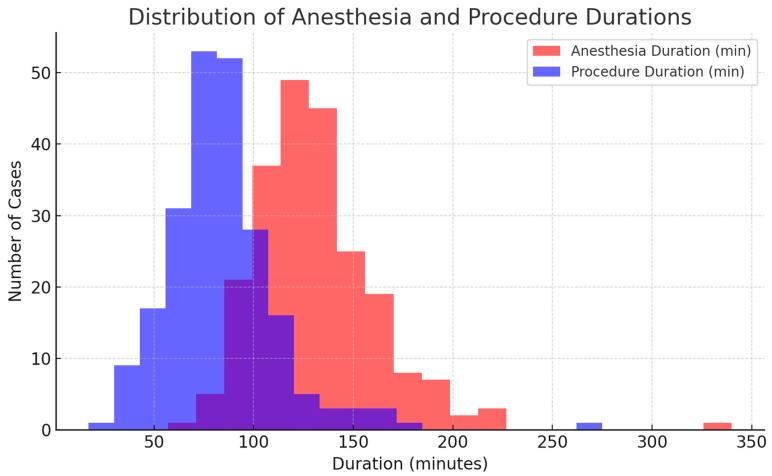
Distribution of anesthesia and procedure durations. This figure presents the distribution of total anesthesia and procedure times among the study patients, highlighting variability in duration across cases. Purple (blue-purple) represents procedure duration (minutes).

**Figure 10:**
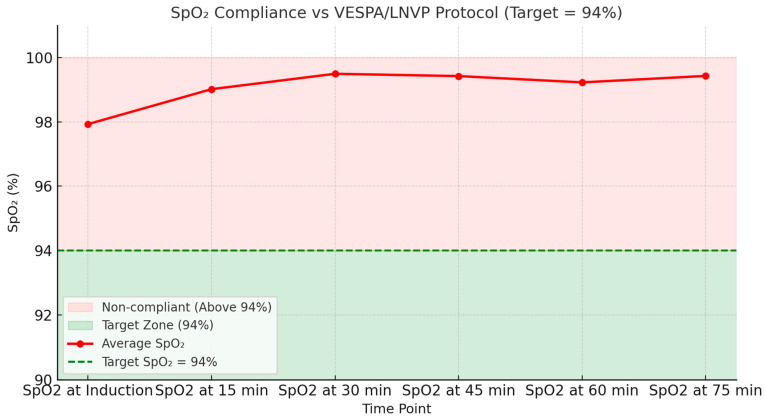
SpO_2_ trend and compliance. Although both the VESPA and LNVP recommendations advise adjusting FiO_2_ to maintain SpO_2_ around 94%, this figure clearly demonstrates that SpO_2_ values remained significantly higher across all time intervals.

Most importantly, analysis of Figure 10 demonstrated no clear physiological correlation between deviations in tidal volume, PEEP, or FiO_2_ and oxygenation targets or procedural outcomes.

Our results also indicated that patients diagnosed with malignancy had a higher mean smoking history (approximately 27 pack-years) compared to those without malignancy (approximately 18 pack-years).

All patients received propofol as the sole induction agent. Those administered higher induction doses of propofol also received higher total doses during the procedure. Sevoflurane was used for maintenance anesthesia, with some patients additionally receiving a low-dose propofol infusion. Fentanyl was the most commonly used adjunct (mean dose ≈ 101 µg; range: 25–300 µg; median and mode: 100 µg). No patients received etomidate, ketamine, or midazolam.

Figure 9 provides information about distribution of anesthesia and procedure durations. The average duration of anesthesia was approximately 131 min (2 h and 11 min), while the mean procedure time was 84 min (1 h and 24 min). On average, anesthesia lasted about 45–50 min longer than the procedure itself, reflecting the expected periods of induction, preparation, and emergence. Of the 224 patients, only one required reintubation following post-procedural extubation. This patient developed acidosis and hypercarbia shortly after extubation and was subsequently reintubated in the procedure room. A pleural effusion was drained, after which the patient was successfully extubated in the recovery area. There were no additional intra- or post-procedural complications. No patient required post-procedural ICU admission, and there were no in-hospital or 30-day mortalities.

Regarding the first biopsy location, a total of 60 biopsies were obtained from the lower lobes—33 from the right lower lobe and 27 from the left lower lobe. The remaining biopsies were from the upper lobes, with 80 from the right upper lobe and 49 from the left upper lobe. In a few cases, the lesion location was not clearly specified. The distribution of biopsy sites during the second attempt was largely similar, and many lesions were noted to be bilateral.

In summary, adherence to the VESPA and LNVP ventilation protocols was limited, with frequent deviations observed in FiO_2_, PEEP, and tidal volume parameters. These findings indicate variable compliance with lung-protective ventilation strategies, reflecting a tendency toward higher oxygen delivery despite already adequate SpO_2_ levels during anesthesia. Despite these inconsistencies, procedural performance remained strong, as evidenced by a high rate of navigation success and diagnostic accuracy. This suggests that while ventilation practices deviated from protocol recommendations, they did not compromise the overall clinical outcomes.

## 4. Discussion

The diagnostic accuracy of navigational bronchoscopy remains variable. In a review, Muñoz-Largacha reported success rates that ranged from 55% to 94% across 13 studies [5,6,7,8,9,10,11,12,13,14,15,16]. A more extensive meta-analysis by Kops et al., which included 95 studies (10,381 patients and 10,682 nodules), demonstrated a pooled diagnostic accuracy of 70.9% (95% CI, 68.4–73.2%) [17]. Notably, pneumothorax occurred in 2.5% of patients.

Several factors have been proposed to account for the variability in diagnostic accuracy, including the influence of anesthesia and positive pressure ventilation. In North America, these procedures are most often performed under general anesthesia with intermittent positive pressure ventilation.

In an effort to enhance diagnostic accuracy, expert groups have proposed specific ventilation strategies, primarily emphasizing the optimization of tidal volume, fraction of inspired oxygen (FiO_2_), and positive end-expiratory pressure (PEEP). The VESPA protocol was briefly explained above. In arriving at this protocol, the authors, Salahuddin et al., enrolled 76 patients (38 per arm) and evaluated atelectasis occurrence by chest CT and Radial Probe Endobronchial Ultrasound (RP-EBUS) [4]. Some of their recommendations include moderate PEEP (8–10 cm H_2_O) and avoiding FiO_2_ = 100% unless absolutely necessary. To administer such high degree of PEEP, patients need to be intubated with an endotracheal tube (laryngeal mask airway is unsuitable). Further, they recommend a lung recruitment maneuver to open collapsed alveoli, soon after induction and intubation. An FiO2 needs to be titrated for a SpO_2_ > 94%, and volume control ventilation should be targeted at ~6–8 mL/kg of ideal body weight. These measures are supposed to reduce atelectasis and help maintain better lung inflation and target visibility, thereby minimizing CT-to-body divergence risk. The net result is better diagnostic accuracy as a result of maintaining better lung architecture during the procedure. We could not find any studies that have evaluated the diagnostic accuracy after VESPA recommendations. However, a trial is in progress [18].

The second anesthesia/ventilation strategy is the Lung Navigation Ventilation Protocol (LNVP). Its core elements mirror the VESPA protocol and include endotracheal intubation (rather than an LMA); a low FiO_2_ (typically 0.30–0.50) titrated to maintain SpO_2_ > 92–94% while avoiding 100% O_2_ except during emergencies; moderate-to-high PEEP (generally 10–15 cm H_2_O); tidal volumes of 6–8 mL/kg ideal body weight using volume-controlled ventilation; and recruitment maneuvers with sustained inflation pressures of 30–40 cm H_2_O for approximately 20–30 s.

Bhadra et al. 2022 compared LNVP vs. conventional ventilation in guided bronchoscopy with CBCT imaging, and found decreased atelectasis and better diagnostic yield (92% vs. 70%, *p* = 0.08), although it did not have statistical power to make definitive conclusions [3].

We would like to draw the reader’s attention to another important terminology used in our study. Contemporary literature, including the Target and Benefit study, defines navigation success in robotic bronchoscopy as the ability of the robotic catheter to reach the target lesion and successfully obtain tissue samples [19]. This definition does not necessitate confirmation of “tool-in-lesion” placement through advanced imaging modalities. The present study adopts this widely accepted definition, which has been consistently applied across multiple early and contemporary robotic bronchoscopy reports.

Radial endobronchial ultrasound (r-EBUS) remains an established and reliable method for confirming target localization, particularly in the hands of experienced operators. Recent studies, including the VESPA trial and others, have demonstrated that r-EBUS imaging provides sufficient confirmation for most solid pulmonary nodules. For pure ground-glass opacities (GGOs), however, radial imaging alone may be inadequate; in such cases, our institutional practice is to supplement navigation with augmented three-dimensional imaging—specifically 3D fluoroscopy rather than cone-beam CT.

Nevertheless, the primary objective of this study was to evaluate anesthetic management during robotic bronchoscopy rather than to analyze diagnostic performance metrics in detail. In alignment with established reporting standards, we have replaced the term “diagnostic yield” with “diagnostic accuracy.” In this study, diagnostic accuracy refers to tissue sampling results that were deemed clinically sufficient—defined as samples that did not require further invasive procedures or that were not contradicted by clinical or radiologic follow-up within one year (e.g., lesion progression or a subsequent surgical diagnosis). Diagnostic accuracy is typically established after more than one year of follow-up, and ideally after two years. In our analysis, all patients were followed for a minimum of one year, and many are monitored well beyond two [20]. It is well recognized that shorter follow-up periods (<1 year) may misclassify slow-growing malignancies as benign, thereby inflating the negative predictive value. Likewise, Folch EE et al., in the landmark NAVIGATE trial, used a 24-month follow-up interval to classify nondiagnostic ENB outcomes as true-negative or false-negative [21].

Our results are consistent with nationally reported outcomes for combined use of shape-sensing robotic bronchoscopy and advanced imaging technologies and do not reflect overestimation. [22,23,24,25,26,27]. Given the retrospective and descriptive design, these findings demonstrate association rather than causation. The analysis was not powered or structured to identify independent predictors of diagnostic accuracy; therefore, conclusions regarding the direct impact of ventilatory strategy should be interpreted cautiously. For completeness, we have also included the lobe distribution of sampled nodules, given its potential relevance to navigation performance and procedural efficiency. As mentioned in results, about one third of the lesions were in the lower lobes. These findings suggest that strict adherence to predefined ventilatory protocols may not be required to achieve acceptable outcomes in experienced high-volume centers. The sole component of the proposed protocols that was consistently applied in our practice was the recruitment maneuver. It is likely that recruitment maneuvers may represent a key component of current protocols. In a review of peri-operative pulmonary atelectasis, it is stated that “pulmonary atelectasis, most frequently located in the dorso-caudal regions, represents an almost constant feature of general anesthesia [28]. A prospective observational study (the I-LOCATE trial) found >50% incidence of atelectasis in *dependent lower-lobe segments* >30 min after induction of anesthesia [29]. Another CT study found that after induction of anesthesia and pneumoperitoneum, atelectasis volume increased in the dependent lung regions. They noted that “both upper and lower lobes reacted the same way,” but the emphasis was on the dependent portions [30]. The classic review by Hedenstierna & Rothen (“Atelectasis formation during anesthesia …”) reported that collapsed lung tissue is present in about 90% of subjects under anesthesia, and although it does not precisely map lobe by lobe, the mechanism points to collapse of dependent lung units [31].

In mechanical ventilation, a recruitment maneuver is designed to reopen collapsed lung units by transiently raising airway pressures, whereas the application of positive end-expiratory pressure (PEEP) serves primarily to maintain lung volume once these units are reopened [32]. This distinction has direct physiological and procedural relevance for bronchoscopic navigation: reopening previously collapsed alveoli may improve ventilatory distribution and airway patency during the procedure, while sustained PEEP helps keep alveoli open, thus reducing dynamic derecruitment and potentially improving procedural visibility and stability. A recruitment maneuver transiently elevates mean airway or transpulmonary pressure to open non-aerated alveoli [33]. Following recruitment, PEEP is applied to prevent collapse of these units during expiration or when ventilatory conditions change [34]. The “opening pressure” for alveoli is often higher than the “keeping open” pressure (i.e., the PEEP), which underscores why recruitment and PEEP are functionally distinct [34].

Periodic application of recruitment maneuvers during lengthy bronchoscopic procedures may help limit CT-to-body divergence by restoring ventilation to lung regions that progressively collapse under anesthesia. By re-expanding these dependent areas, repeated recruitment can help preserve lung volume and maintain closer alignment between the pre-procedural CT model and the patient’s real-time thoracic anatomy. Physiologically, this is consistent with the known role of recruitment in reopening non-aerated alveoli, enhancing uniformity of aeration, and stabilizing lung structure—factors that may collectively reduce the degree of anatomical shift observed over time. Prior studies in anesthetized and critically ill populations have demonstrated that such maneuvers are effective in reversing atelectasis and restoring aerated lung volume [35]. The VESPA ventilation strategy is intended to minimize anesthesia-related atelectasis, which can distort airway anatomy and increase CT-to-body divergence during navigational bronchoscopy across all lung regions. While it is true that anesthesia-induced atelectasis occurs predominantly in dependent lung zones—most commonly the posterior and lower lobes—this simply means that these areas derive the greatest benefit from the strategy; it does not imply that the VESPA protocol or its recommendations are limited to lower-lobe lesions.

The lack of strict adherence these strategies in totality in our practice may reflect limited familiarity with the protocols and the lack of dedicated anesthesia teams routinely assigned to the bronchoscopy suite.

### Limitations

This study has several limitations. The retrospective single-center design limits generalizability and introduces potential selection and documentation bias. The descriptive analytic approach without multivariable modeling prevents identification of independent predictors of diagnostic success or ventilatory effects. Additionally, not all benign lesions received definitive etiologic classification when clinical management did not require further investigation. The absence of formal hypothesis testing limits statistical inference and causal interpretation.

## 5. Conclusions

In this retrospective study we observed high navigational success (89.2%) and good diagnostic accuracy (81.7%), despite limited adherence to the predefined VESPA and LNVP ventilation protocols. The frequent deviations in FiO_2_, PEEP, and tidal volume indicate variable compliance with lung-volume-preservation and atelectasis-prevention strategies. Nonetheless, our outcomes remained favorable relative to published benchmarks, likely reflecting the consistent use of recruitment maneuvers, the high procedural volume, and the specialized expertise at our center. These findings suggest that specific key elements—particularly recruitment maneuvers—may contribute more substantially to procedural success than strict protocol adherence. It is also important to recognize that certain ventilatory adjustments recommended in these protocols, such as higher levels of PEEP or lower FiO_2_ targets, may pose risks in select patients by reducing physiological reserve.

Prospective studies are needed to confirm these observations and delineate the relative importance of individual ventilatory components. Importantly, our findings suggest that acceptable navigational success and diagnostic accuracy may be achievable without strict adherence to predefined ventilatory protocols, particularly in experienced high-volume centers. Future research should focus on identifying which specific components of ventilation strategies provide the greatest clinical benefit and whether targeted application based on lesion location or patient characteristics offers a more effective approach than universal protocolization. 

## Figures and Tables

**Table 1:** Baseline Characteristics of Patient Population.

Total Number of Bronchoscopy Procedures	224
Demographics	
#	
Male	88
Female	136
(Median (IQR))	
Height (m)	1.65 (0.15)
Weight (kg)	69.7 (23.8)
BMI	25.1 (6.8)
Clinical Characteristics (#)	
ASA Status	
II	33
III	168
IV	23
Hypertension	114
CAD	30
Ischemic Heart Disease	11
Pulmonary Hypertension	9
Obstructive Sleep Apnea	30
History of Bronchoscopy	32

**Table 2:** Procedure and Anesthesia Information. Values are presented as Mean ± SD, N (%), or Median (LQ, UQ).

Bronchoscopy Location	
Location	N (%)
Right	91 (40.0%)
Left	49 (21.8%)
Bilateral	84 (37.3%)
Anesthesia Information	
Parameter	Value
Propofol Induction Dose (mg)	150 (120, 200)
Total Propofol Dose (mg)	180 (140, 200)
Total Fentanyl Dose (µg)	100 (100, 100)
Total Anesthesia Time (min)	127 (111, 146)
Total Procedure Time (min)	82 (68, 96)
Endotracheal Tube Size (mm)	
Size (mm)	N (%)
7.5	8 (3.6%)
8.0	9 (4.0%)
8.5	202 (90.1%)

**Table 3:** Final Diagnostic Classification and Outcome Performance Metrics. N = 213.

Final Classification	Diagnostic Criteria (Initial + 2-Year Follow-Up)	Total (N)	Percentage (%)
True Positive	Diagnosis indicated malignancy (e.g., “Adenocarcinoma”, “Carcinoma”, “Suspicious for malignancy”) and was confirmed accurate at 2 years.	95	44.6%
True Negative (Specific)	Diagnosis indicated a specific benign etiology (e.g., “Granuloma”, “Infection”, “Grew MAC”, “Organizing Pneumonia”) and was confirmed accurate at 2 years.	45	21.12%
True Negative (Non-Specific)	Diagnosis was negative for malignancy but lacked a specific benign diagnosis (e.g., “Inflammation”, “Negative”, “Fibrosis”) and was confirmed accurate (patient was truly disease-free) at 2 years.	34	15.96%
False Positive	Diagnosis indicated malignancy, but the final diagnosis determined that the patient actually had benign disease.	4	1.88%
False Negative	Diagnosis was benign (specific or nonspecific) or negative, but the patient was later found to have malignancy.	12	5.63%
Non-Diagnostic	The procedure yielded insufficient material for diagnosis (e.g., “Nondiagnostic”, “Hypocellular”, “Insufficient”)	23	10.8%

**Table 4:** Location of the lesions.

Characteristic	Median (IQR Range), N (%)
**Mean Lesion Diameter (cm)**	2.18
**Lesion Location**	N = 222
- Right Upper Lobe	1.9 (1.3, 2.70), N = 81 (36.4%)
- Right Middle Lobe	2.4 (1.5, 3.00), N = 21 (9.5%)
- Right Lower Lobe	1.7 (1.2, 2.4), N = 41 (18.5%)
- Left Upper Lobe	2.00 (1.3, 2.7), N = 50 (22.5%)
- Left Lower Lobe	1.7 (1.2, 3.00), N = 25 (11.3%)

**Table 5:** Body Mass Index of patients included in the study. This table summarizes the BMI values of all patients analyzed, categorized according to standard WHO classifications.

Category	BMI Range (kg/m^2^)	% of Patients
Underweight	<18.5	12.0%
Normal	18.5–24.9	35.6%
Overweight	25–29.9	33.2%
Obese Class I	30–34.9	10.1%
Obese Class II	35–39.9	4.3%
Obese Class III	≥40	4.8%

## Data Availability

The datasets generated and/or analyzed during the current study are not publicly available due to patient privacy and institutional restrictions but are available from the corresponding author on reasonable request and with appropriate institutional approval.
